# Development and Validation of a Deep Learning–Based Synthetic Bone-Suppressed Model for Pulmonary Nodule Detection in Chest Radiographs

**DOI:** 10.1001/jamanetworkopen.2022.53820

**Published:** 2023-01-31

**Authors:** Hwiyoung Kim, Kye Ho Lee, Kyunghwa Han, Ji Won Lee, Jin Young Kim, Dong Jin Im, Yoo Jin Hong, Byoung Wook Choi, Jin Hur

**Affiliations:** 1 Department of Radiology and Research Institute of Radiological Science and Center for Clinical Image Data Science, Severance Hospital, Yonsei University College of Medicine, Seoul, Korea; 2 Department of Radiology, Dankook University Hospital, Cheonan, Chungnam Province, Republic of Korea; 3 Department of Radiology, Pusan National University Hospital, Pusan National University School of Medicine, Busan, Korea; 4 Medical Research Institute, Busan, Korea; 5 Department of Radiology, Dongsan Medical Center, Keimyung University College of Medicine, Daegu, Korea

## Abstract

**Importance:**

Dual-energy chest radiography exhibits better sensitivity than single-energy chest radiography, partly due to its ability to remove overlying anatomical structures.

**Objectives:**

To develop and validate a deep learning–based synthetic bone-suppressed (DLBS) nodule-detection algorithm for pulmonary nodule detection on chest radiographs.

**Design, Setting, and Participants:**

This decision analytical modeling study used data from 3 centers between November 2015 and July 2019 from 1449 patients. The DLBS nodule-detection algorithm was trained using single-center data (institute 1) of 998 chest radiographs. The DLBS algorithm was validated using 2 external data sets (institute 2, 246 patients; and institute 3, 205 patients). Statistical analysis was performed from March to December 2021.

**Exposures:**

DLBS nodule-detection algorithm.

**Main Outcomes and Measures:**

The nodule-detection performance of DLBS model was compared with the convolution neural network nodule-detection algorithm (original model). Reader performance testing was conducted by 3 thoracic radiologists assisted by the DLBS algorithm or not. Sensitivity and false-positive markings per image (FPPI) were compared.

**Results:**

Training data consisted of 998 patients (539 men [54.0%]; mean [SD] age, 54.2 [9.82] years), and 2 external validation data sets consisted of 246 patients (133 men [54.1%]; mean [SD] age, 55.3 [8.7] years) and 205 patients (105 men [51.2%]; mean [SD] age, 51.8 [9.1] years). Using the external validation data set of institute 2, the bone-suppressed model showed higher sensitivity compared with that of the original model for nodule detection (91.5% [109 of 119] vs 79.8% [95 of 119]; *P* < .001). The overall mean of FPPI with the bone-suppressed model was reduced compared with the original model (0.07 [17 of 246] vs 0.09 [23 of 246]; *P* < .001). For the observer performance testing with the data of institute 3, the mean sensitivity of 3 radiologists was 77.5% (95% [CI], 69.9%-85.2%), whereas that of radiologists assisted by DLBS modeling was 92.1% (95% CI, 86.3%-97.3%; *P* < .001). The 3 radiologists had a reduced number of FPPI when assisted by the DLBS model (0.071 [95% CI, 0.041-0.111] vs 0.151 [95% CI, 0.111-0.210]; *P* < .001).

**Conclusions and Relevance:**

This decision analytical modeling study found that the DLBS model was more sensitive to detecting pulmonary nodules on chest radiographs compared with the original model. These findings suggest that the DLBS model could be beneficial to radiologists in the detection of lung nodules in chest radiographs without need of the specialized equipment or increase of radiation dose.

## Key Points

**Question:**

Can a deep learning–based synthetic bone-suppressed (DLBS) model additionally improve the detection of pulmonary nodules on chest radiographs?

**Findings:**

In this decision analytical modeling study of 1449 patients, the DLBS model was more sensitive to detecting pulmonary nodules on chest radiographs compared with the original model. In addition, radiologists experienced improved nodule-detection performance when assisted by the DLBS model.

**Meaning:**

These results suggest that the DLBS model could be beneficial to radiologists in the detection of lung nodules in chest radiographs without need of the specialized equipment or increase of radiation dose.

## Teaser

This decision analytical modeling study aims to develop and validate a deep learning–based synthetic bone-suppressed nodule-detection algorithm for pulmonary nodule detection on chest radiographs.

## Introduction

Chest radiography is the most commonly performed diagnostic imaging procedure, which is used for screening, diagnostic workups, and monitoring of various thoracic diseases.^1,2^ However, many prior studies have indicated the limitations of chest radiography for lung cancer screening with a low detection rate, especially for small pulmonary nodules.^3,4^ Therefore, accurate interpretation of chest radiographs requires a great deal of experience and medical knowledge on the part of the radiologist. In addition, chest radiography is subject to substantial interreader variability and suboptimal sensitivity for important clinical findings.

Although chest radiography is clinically useful, efficient, and cost-effective, this examination consists of complex 3-dimensional anatomic information condensed in a 2-dimensional projection. Therefore, small nodules may be shielded by the ribs and scapula and thus missed during interpretation of the x-ray. To solve this issue, dual-energy subtraction techniques were developed to distinguish bone from soft tissue. Dual-energy chest radiography (DECR) exhibits better sensitivity than single-energy chest radiography, partly due to its ability to remove overlying anatomical structures. DECR has been demonstrated to improve the ability to detect and characterize lung nodules^5,6,7,8^; however, disadvantages of DECR include the requirement of specialized equipment and a small potential increase in radiation dose.

Deep learning technology has the potential to automatically detect abnormalities or assist radiologists in reading chest radiographs. Several artificial intelligence algorithms have been tested in an effort to reduce radiologist errors and increase the detection rate of pulmonary nodules on chest radiographs.^9,10,11,12^ In addition, several studies have focused on bone-suppression techniques using artificial intelligence.^13,14,15^

A convolutional neural network (CNN) is an artificial intelligence technique that has been widely applied to date in the medical field and can perform various tasks such as image classification, segmentation, and regression with high accuracy. We developed a deep learning–based synthetic bone-suppressed (DLBS) pulmonary nodule-detection algorithm by modifying a conventional U-net to take advantage of the high frequency-dominant information that propagates from the encoding part to the decoding part. The presented network is different from conventional deep learning–based image processing, which has been demonstrated to improve image characteristics, such as noise and resolution. The main idea of the developed model is that when a feature is propagated from encoding to decoding, only the high frequency components are extracted and propagated. The proposed model also dramatically reduces the number of parameters by adding features of the encoding that propagate to the decoding part instead of the feature concatenation of U-net.^16^

The purpose of this study was to develop and validate a DLBS nodule-detection algorithm for the detection of pulmonary nodules on chest radiographs and to compare detection performance with that of thoracic radiologists.

## Methods

For this decision analytical modeling study, ethics review and institutional review board approval were obtained from all participating institutions (Severance Hospital, Pusan National University Hospital, and Dongsan Medical Center) and the requirement for informed consent was waived because of this retrospective study design. The posterior-anterior projection chest radiographs obtained from 3 tertiary hospitals were collected for the development and validation of a DLBS nodule-detection algorithm. All chest radiographs were deidentified. The candidate radiographs were sorted by inclusion and exclusion criteria, regardless of the type of acquisition system (computed radiography or digital radiography) or the manufacturer of the radiography device. This study followed the relevant portions of the Consolidated Health Economic Evaluation Reporting Standards (CHEERS) reporting guideline.

### Data Sets

For algorithm development, we retrospectively collected 1004 chest radiographs obtained between November 2015 and December 2019 from a single center (institution 1). Normal radiographs were included from healthy adults (aged at least 19 years) who underwent health screening chest radiographs and, if a normal chest computed tomography (CT) scan was also performed within 14 days of the radiograph, it was collected.

Chest radiographs with lung nodules were collected based on the following inclusion criteria: (1) adult patients (aged at least 19 years) who underwent biopsy or surgery for 1 or more CT- and pathology-proven lung nodules, regardless of pathologic type (malignant neoplasm or benign) and component (solid, partly solid, or ground glass); (2) at least 1 nodule needed to be pathologically proven; (3) the number of lesions per radiograph was 3 or fewer; (4) all nodules on the radiograph measured between 1 to 3 cm in diameter on CT imaging (the short-axis length on any CT plane was used to avoid overestimation); and (5) the nodules were not in a major airway or the mediastinum. Chest radiographs containing abnormal findings other than lung nodules, such as consolidation, atelectasis or pleural effusion, were not excluded. Any radiographs considered unsuitable for clinical interpretation were excluded. All chest radiographs were carefully reviewed by 2 experienced thoracic radiologists based on consensus.

By using the aforementioned criteria, the chest radiography data were randomly assigned into 1 of the following 3 data sets: a training data set that consisted of 800 chest radiographs (including 335 normal and 465 nodule chest radiographs), a tuning data set (composed of 98 chest radiographs consisting of 48 normal and 50 nodule chest radiographs), and an internal validation data set (composed of 100 chest radiographs consisting of 40 normal and 60 nodule chest radiographs) to validate the detection performance of the trained network (eFigure 1 in the Supplement). Two additional independent data sets were prepared for external validation from 2 different hospitals (institute 2, 246 patients with 131 normal and 115 nodule chest radiographs; institute 3, 205 patients with 113 normal and 92 nodule chest radiographs) using the same inclusion and exclusion criteria (eFigure 1 in the Supplement). Detailed demographic information is provided in Table 1.

**Table 1.** Baseline Characteristics of Data Sets

Variable	No. (%)		
	Institute 1 (n = 998)	Institute 2 (n = 246)	Institute 3 (n = 205)
Sex			
Male	539 (54.0)	133 (54.1)	105 (51.2)
Female	459 (46.0)	113 (45.9)	100 (48.8)
Age, mean (SD), y	54.2 (9.82)	55.3 (8.74)	51.8 (9.13)
Normal radiographs	423 (42.4)	131 (53.2)	113 (54.9)
Nodule chest radiographs	575 (57.6)	115 (46.8)	92 (44.8)
Total No. of nodules	598	119	92
Nodule size on CT	23.8 (8.8)	24.4 (7.4)	25.1 (8.7)
mean (SD), mm			
1.0 cm ≤ × < 2.0 cm	198 (33.1)	49 (41.2)	28 (30.4)
2.0 cm ≤ × < 3.0 cm	400 (66.9)	70 (58.8)	64 (69.6)
Location			
Right			
Upper lobe	160 (26.7)	30 (25.2)	25 (27.1)
Middle lobe	36 (6.0)	11 (9.2)	10 (10.9)
Lower lobe	131 (21.9)	24 (20.2)	22 (24.0)
Left			
Upper lobe	142 (23.7)	29 (24.4)	25 (27.1)
Lower lobe	129 (21.7)	25 (21.0)	10 (10.9)
Internal characteristics of CT findings			
Subsolid nodule	32 (5.4)	5 (4.2)	3 (3.3)
Solid nodule	566 (94.6)	114 (95.8)	89 (96.7)
Pathology			
Benign	65 (10.9)	18 (15.1)	10 (10.9)
Malignant (primary)	483 (80.7)	95 (79.8)	74 (80.4)
Malignant (metastatic)	50 (8.4)	6 (5.1)	8 (8.7)

Abbreviation: CT, computed tomography.

### Labeling and Annotation (Standard Reference)

In the developmental data sets, chest radiographs were labeled as either normal or nodule chest radiographs (image-level labeling), and the locations of nodules on the nodule chest radiographs were annotated. Two thoracic radiologists (with more than 5 years of experience) reviewed each CT scan as a standard of reference and marked the location of true nodule(s) as a region of interest (ROI) in consensus. Chest CT scans performed within 2 weeks were used as references. For the 2 external validation data sets, 2 thoracic radiologists also labeled the chest radiographs and annotated the location of the nodules on chest radiographs on the basis of the chest CT scans performed within 2 weeks.

### Development of the DLBS Model

The developed deep learning–based model consisted of 2 subsystems responsible for (1) generating bone- and soft tissue–only images from single-energy chest radiography and (2) detecting suspicious pulmonary nodules, respectively.

For the first step, we previously developed a deep convolutional neural network (DCNN)-based synthetic bone-suppressed algorithm based on U-net,^16^ which is a deep convolutional neural network architecture with multiresolution analysis performed by repeated convolution and feature-dimension changes. The main idea of the developed model was that, when a feature is propagated from encoding to decoding, only the high-frequency components are extracted and propagated. The model selectively projected the bone- and soft tissue–only chest radiography images from a single energy chest radiography image. The bone-suppressed chest radiographs were automatically synthesized through a DCNN-based encoder-decoder model. A detailed description of the development of the deep learning–based algorithm can be found in a previous study.^16^

**Figure 1.**
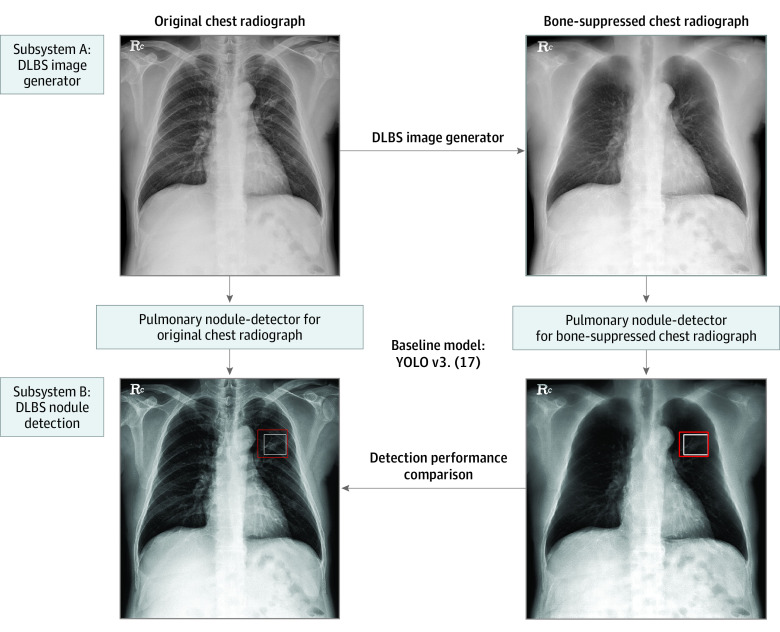
Schematic of the Developed Deep Learning–Based Model The model consisted of 2 subsystems responsible for (1) generating bone-suppressed images from single-energy chest radiography and (2) detecting suspicious pulmonary nodules. DLBS indicates deep learning–based synthetic bone-suppressed; YOLO, you only look once.

For the second step, we developed a pulmonary nodule–detection algorithm based on a convolution neural network (CNN) algorithm known as “you only look once” (YOLO).^17^ The developed algorithm customized a YOLO version 3 CNN algorithm for the detection of pulmonary nodules. In general, the network consists of 2 major components: (1) a feature extractor that screens nodule presence among the input data, and (2) a bounding box generator that determines nodule location. The DLBS model was trained to detect lung nodules using the training data set involving synthetic bone-suppressed images and the CNN model was separately trained to detect lung nodules using original chest radiographs (Figure 1; and eFigure 2 in the Supplement). To maximize the nodule-detection performance, an ensemble model was developed through 5-fold cross-validation, and a hard-negative sampling method was used (ensemble model).

The DLBS model used automatic synthetic bone–suppression images to detect pulmonary nodules from original chest radiographs, whereas the CNN model detected pulmonary nodules on original chest radiographs.

### Evaluation of the DLBS and CNN Models

**Figure 2.**
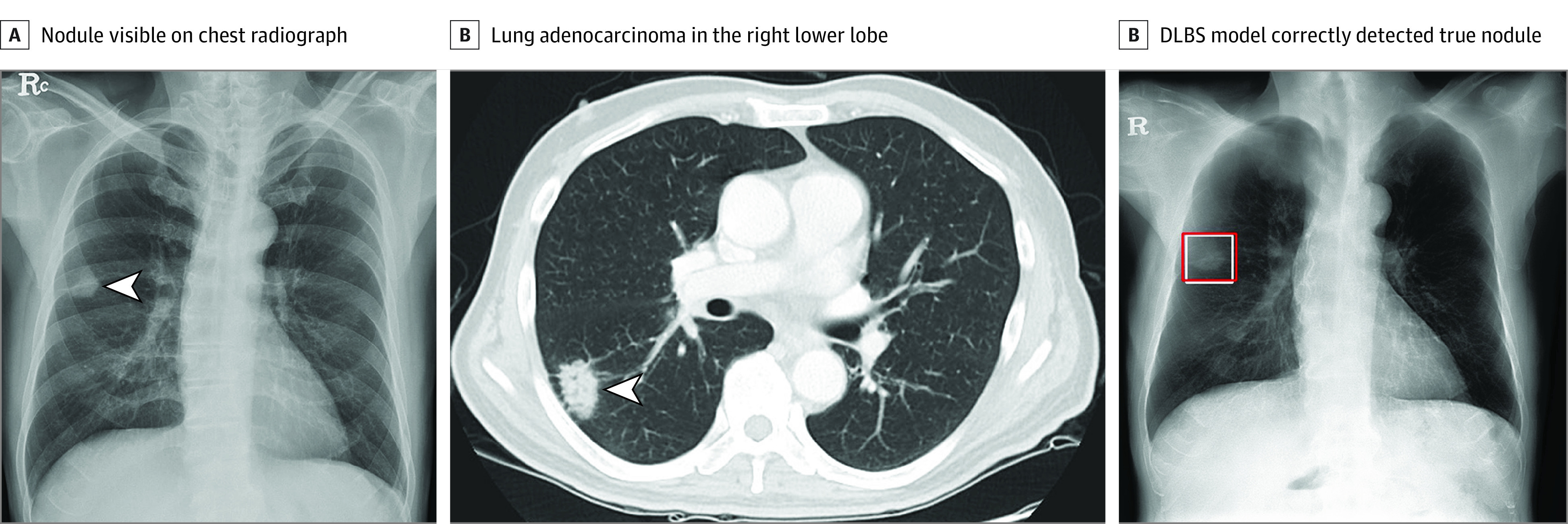
Illustration of Representative Case of Nodule Detection Performance of Deep Learning–Based Synthetic Bone-Suppressed (DLBS) Nodule-Detection Algorithm Chest radiograph images of a man aged 59 years with primary adenocarcinoma. A, The nodule was visible on the chest radiograph (arrow). B, Chest CT examination revealed a 27-mm lung adenocarcinoma in the right lower lobe (arrow). C, On bone-suppressed chest radiographs created using the DLBS model, the algorithm correctly detected the true nodule (white box: ground truth, red box: DLBS).

For internal (institute 1) and external validation (institutes 2 and 3), the nodule-detection performances were evaluated on a per-nodule basis. First, we evaluated the performance of the DLBS nodule-detection algorithm (DLBS model) compared with that of the CNN nodule-detection algorithm (CNN model) using internal (institute 1) and external validation (institute 2 and institute 3) data sets (Figure 2). We compared the nodule-detection performance of the CNN nodule-detection algorithm (CNN model) trained with the original chest radiographs and their corresponding bone-suppressed chest radiographs (DLBS model), respectively.

Second, an observer performance test was conducted by using the data sets from institute 3 to compare the nodule-detection performance of the DLBS model with that of the physicians. During test 1, 3 thoracic radiologists (with more than 5 years of experience) independently reviewed each chest radiograph to discriminate normal chest radiographs from nodule chest radiographs and localized lung nodules (nodule detection) without using the DLBS model. The readers independently analyzed chest radiographs without clinical information, prior radiographs, or CT findings, then marked up to 3 regions with individual ROIs that were suspicious for nodules. The readers knew that each image can have up to 3 nodules (0-3), but they did not know which radiographs were normal or had nodules.

During test 2, each reader reanalyzed the same images from test 1 assisted with the DLBS model after washout period of 1 week from test 1. The readers reanalyzed chest radiographs assisted with the detection results from the DLBS model at the same time. Each reviewer was then asked to mark up to 3 regions with individual ROIs if any nodule was suspected in the image.

### Statistical Analysis

The data result was presented in a binary format in which the presence and location of nodule is displayed in a bounding box. Sensitivity was defined as the number of true-positive markings divided by the number of ground-truth ROIs and compared using logistic regression. A projected box was considered a true positive if the box covered more than 50% of the area of the ground truth box. False-positive markings per image (FPPI) were defined as the total number of false-positive markings divided by the total number of radiographs and compared with Poisson regression. The generalized estimating equation was applied to account for clustering effects caused by the multicenter and/or multireader design.

*P* < .05 was considered statistically significant. Statistical analyses were performed from March to December 2021 using the SAS version 9.4 software program (SAS Institute) and R version 4.1.13 statistical package (R Project for Statistical Computing).

## Results

### Study Participants

Training data consisted of 998 patients (539 men [54.0%]; mean [SD] age, 54.2 [9.82] years) from institute I. There were 598 nodules in the 575 nodule chest radiographs. The mean (SD) nodule size measured from CT images was 23.8 (8.8) mm. A total of 80.7% (483 of 598) nodules were primary lung cancers, 8.4% (50 of 598) were metastases, and 10.9% (65 of 598) were benign. Two external validation data sets consisted of 246 patients (133 men [54.1%]; mean [SD] age, 55.3 [8.74] years) and 205 patients (105 men [51.2%]; mean [SD] age, 51.8 [9.13] years) were used to validate DLBS nodule-detection performance, respectively. There were 119 nodules with a mean (SD) size of 24.4 (7.4) mm from institute 2 and 92 nodules with a mean (SD) size of 25.1 (8.7) mm from institute 3. The demographic characteristics of participants for the nodule data sets are summarized in Table 1.

### Nodule Detection Performance of the DLBS and CNN Models

For the internal validation data set of 100 chest radiographs (40 normal and 60 nodule chest radiographs), our original model (the CNN algorithm) showed a sensitivity of 86.7% (52 of 60) for nodule-detection performance. When the bone-suppressed model (the DLBS model) independently analyzed chest radiographs, the sensitivity was improved compared with the original model (96.7% [58 of 60] vs 86.7% [52 of 60]; *P* = .008) (eFigure 3 in the Supplement). The rates of FPPI with the original model was 0.06 (6 of 100) and the bone-suppressed model was 0.05 (5 of 100). The overall mean of FPPI with the original model was not significantly different compared with that of FPPI with the bone-suppressed model (*P* = .71) (Table 2).

**Table 2.** Nodule-Detection Performance of Bone-Suppressed DCNN Model in the Internal and External Validation Tests

Variables	Original model	DLBS model	*P* value
Institute 1 (n = 100)			
Sensitivity, % (No./total No.)	86.7 (52/60)	96.7 (58/60)	.008
95% CI	78.1-95.3	92.1-100	
FPPI	0.06 (6/100)	0.05 (5/100)	.71
95% CI	0.02-0.15	0.02-0.12	
Institute 2 (n = 246)			
Sensitivity, % (No./total No.)	79.8 (95/119)	91.5 (109/119)	<.001
95% CI	72.7–87.1	87.6-97.2	
FPPI	0.09 (23/246)	0.07 (17/246)	<.001
95% CI	0.06-0.14	0.04-0.11	
Institute 3 (n = 205)			
Sensitivity, % (No./total No.)	80.4 (74/92)	92.4 (85/92)	<.001
95% CI	74.1-90.3	87.0-97.8	
FPPI	0.16 (32/205)	0.09 (19/205)	<.001
95% CI	0.101-0.215	0.05-0.15	

Abbreviations: DCNN, deep convolutional neural network; DLBS, deep learning–based synthetic bone-suppressed; FPPI, false-positive findings per image.

Using external validation data of institute 2 and institute 3, the bone-suppressed model showed a higher sensitivity compared with that of the original model for nodule detection (institute 2: 91.5% [109 of 119] vs 79.8% [95 of 119]; *P* < .001; and institute 3: 92.4% [85 of 92] vs 80.4% [74 of 92]; *P* < .001). The overall mean of FPPI with the bone-suppressed model was reduced compared with that with the original model (institute 2: 0.07 [17 of 246] vs 0.09 [23 of 246]; *P* < .001; and institute 3: 0.09 [19 of 205] vs 0.16 [32 of 205], *P* < .001) (Table 2).

### Reader Performance Test With or Without the DLBS Model

For the observer performance test using institute 3, the 3 radiologists had improved sensitivity using the DLBS model (observer 1: 92.4% [85 of 92] vs 80.4% [74 of 92]; *P* = .001; observer 2: 91.4% [85 of 92] vs 76.1% [70 of 92]; *P* < .001; and observer 3: 91.4% [85 of 92] vs 77.2% [71 of 92]; *P* < .001). The mean sensitivity of the 3 radiologists by themselves was 77.5% (95% CI, 69.9%-85.2%), whereas that of the radiologists when assisted by the DLBS model was 92.1% (95% CI, 86.3%-97.3%; *P* < .001) (Table 3).

**Table 3.** Nodule-Detection Performance of Observer With or Without Bone-Suppressed DCNN Model

Observer	Sensitivity, % (No./total No.)		*P* value	FPPI (No./total No.)		*P* value
	Observer only	Observer + DLBS		Observer only	Observer + DLBS	
Observer 1	80.4 (74/92)	92.4 (85/92)	.001	0.143 (30/205)	0.059 (12/205)	<.001
95% CI	72.3-88.5	87.1-97.8		0.098-0.208	0.034-0.101	
Observer 2	76.1 (70/92)	91.4 (85/92)	<.001	0.165 (35/205)	0.087 (18/205)	.001
95% CI	66.4-83.8	85.7-97.1		0.116-0.235	0.051-0.148	
Observer 3	77.2 (71/92)	91.4 (85/92)	<.001	0.154 (33/205)	0.063 (13/205)	<.001
95% CI	68.6-85.8	85.7-97.1		0.109-0.218	0.051-0.148	
Mean	77.5	92.1	<.001	0.151	0.071	<.001
95% CI	69.9-85.2	86.3-97.3		0.111-0.210	0.041-0.111	

Abbreviations: DCNN, deep convolutional neural network; DLBS, deep learning–based synthetic bone-suppressed; FPPI, false-positive findings per image.

The 3 radiologists achieved a reduced number of FPPI when they were assisted by the DLBS model (observer 1: 0.059 [12 of 205] vs 0.143 [30 of 205]; *P* < .001; observer 2: 0.087 [18 of 205] vs 0.165 [35 of 205]; *P* = .001; and observer 3: 0.063 [13 of 205] vs 0.154 [33 of 205]; *P* < .001) (Table 3). The 3 radiologists had a reduced number of FPPI when assisted by the DLBS model (0.071 [95% CI, 0.041-0.111] vs 0.151 [95% CI, 0.111-0.210]; *P* < .001) (Table 3).

## Discussion

This study was designed to develop and validate whether a DLBS model can additionally improve the detection of pulmonary nodules on chest radiographs and enhance the diagnostic performance of thoracic radiologists. The main finding was that our bone-suppressed model (the DLBS model) could more accurately detect pulmonary nodules on chest radiographs compared with the original model (the CNN algorithm). In addition, radiologists experienced improved nodule-detection performance when assisted by the DLBS model.

Chest radiographs are widely used for the detection of a wide range of lung abnormalities, including pulmonary nodules, but pulmonary nodules can be difficult to detect due to overlap with normal anatomic structures, such as the ribs and clavicle. Use of dual-energy soft-tissue images can improve the detection of focal soft-tissue opacities, such as lung nodules, that may be partly obscured by overlying bony structures.^5,6,7,8^ Previous studies have reported that dual-energy subtraction radiographs improved the detection of lung nodules and masses in daily clinical practice.^6,7,18^ A previous study found that the observer’s performance was further improved by the use of dual-energy soft tissue images (area under the receiver-operating characteristic curve [AUC] from 0.867 to 0.916), and this improvement was statistically significant for the 6 experienced observers (AUC from 0.894 to 0.945).^19^ Despite the advantages, a very limited number of hospitals use dual-energy subtraction radiography because specialized equipment for obtaining dual-energy x-ray exposures is required. In addition, the radiation dose can be increased.

We assumed that our DLBS algorithm could generate lung parenchymal images while subtracting the overlying bony structures from chest radiograph images and therefore efficiently detect lung nodules from lung parenchymal images as the overlying bony structures had already been subtracted. Currently, several deep learning–based algorithms have been tested in an effort to improve nodule-detection performance and reduce radiologist errors on chest radiographs.^11,12,20,21,22,23^ A previous study found that the sensitivity of nodule-detection performances of deep learning–based algorithms ranged from 69.9% to 82.0% with FPPI ranging 0.02 to 0.34 on 4 external validation data sets. In addition, all physicians showed improved nodule-detection performances when assisted by this algorithm.^11^

When we tested the DLBS algorithm, the bone-suppressed model (DLBS model) showed higher sensitivity compared with that of the original model (CNN algorithm) for nodule detection on chest radiographs in external validation data sets (91.5% [109 of 119] vs 79.8% [95 of 119]; *P* < .001; and 92.4% [85 of 92] vs 80.4% [74 of 92]; *P* < .001). In addition, 3 radiologists showed improved sensitivity of nodule detection when assisted by DLBS algorithm (92.1% [95% CI, 86.3%–97.3%] vs 77.5% [95% CI, 69.9%-85.2%]; *P* < .001), and their FPPI decreased from 0.151 to 0.071. A recent study found that the use of generative adversarial networks (GAN)-based bone suppression model with chest radiographs showed comparable nodule detection performance to dual-energy technique in detecting pulmonary nodules on chest radiographs (area under the alternative free-response ROC [AUAFROC] 0.958 vs 0.976; *P* = .35).^23^ This result suggested that GAN-based bone suppression model can improve pulmonary nodule detection performance on chest radiographs. Although this study used GAN-based model to generate automatic bone-suppressed chest radiographs and we used DCNN-based encoder-decoder model, we think the study results showed similar trends. It is difficult to directly compare the lung nodule detection performance of the DLBS model and other commercialized models. However, if we refer to the result of a previous study, the sensitivity of the commercialized nodule detection algorithm (Lunit) was 86.2% (95% CI, 77.8%-94.6%) using a subset of 577 images from 5485 participants, which is similar to the performance of our CNN model.^22^ Therefore, our DLBS model could be beneficial to radiologists in the detection of lung nodules in chest radiographs.

### Limitations

This study has some limitations. First, since the algorithm was validated using retrospective data sets, the possibility of selection bias cannot be excluded. We tried to achieve clinical importance by including 3 different data sets. Second, the high ratio of abnormal to normal radiographs differs from that of clinical practice. As disease prevalence can differ vastly between study populations, our results may not apply in other clinical settings. Third, other lung diseases, such as pneumonia, interstitial lung disease, and pleural effusion, were not considered. Further research is warranted to determine the applicability of this synthetic bone-suppressed model in a prospective multi-institutional study.

## Conclusions

This decision analytical modeling study found that the DLBS model was associated with improved sensitivity for nodule detection compared with the original model on chest radiographs. In addition, these findings suggest that radiologists can improve their nodule-detection performance when assisted by a DLBS model.

## Notes

Supplement. **eFigure 1.** Flowchart of Radiograph Selection **eFigure 2.** The Detailed Architecture of Deep Learning–Based Synthetic Bone-Suppressed Image Generator **eFigure 3.** Illustration of Representative Case of Nodule Detection Performance Between DLBS Model and CNN Model
