# Supplementary material for: Development and Validation of a Deep Learning–Based Synthetic Bone-Suppressed Model for Pulmonary Nodule Detection in Chest Radiographs
Source: JAMA Netw Open. 2023 Jan 31;6(1):e2253820. doi: 10.1001/jamanetworkopen.2022.53820 (PMC9890286; doi:10.1001/jamanetworkopen.2022.53820)
Supplement: Supplement. — eFigure 1. Flowchart of Radiograph Selection eFigure 2. The Detailed Architecture of Deep Learning–Based Synthetic Bone-Suppressed Image Generator eFigure 3. Illustration of Representative Case of Nodule Detection Performance Between DLBS Model and CNN Model [file jamanetwopen-e2253820-s001.pdf]

Supplemental Online Content

Kim H, Lee KH, Han K, et al. Development and validation of a deep learning–based synthetic bone-suppressed model for pulmonary nodule detection in chest radiographs. *JAMA Netw Open.* 2023;6(1):e2253820. doi:10.1001/jamanetworkopen.2022.53820

eFigure 1. Flowchart of Radiograph Selection

eFigure 2. The Detailed Architecture of Deep Learning–Based Synthetic Bone-Suppressed Image Generator

eFigure 3. Illustration of Representative Case of Nodule Detection Performance Between DLBS Model and CNN Model

This supplemental material has been provided by the authors to give readers additional information about their work.

© 2023 Kim H et al. *JAMA Network Open.*

eFigure 1. Flowchart of Radiograph Selection

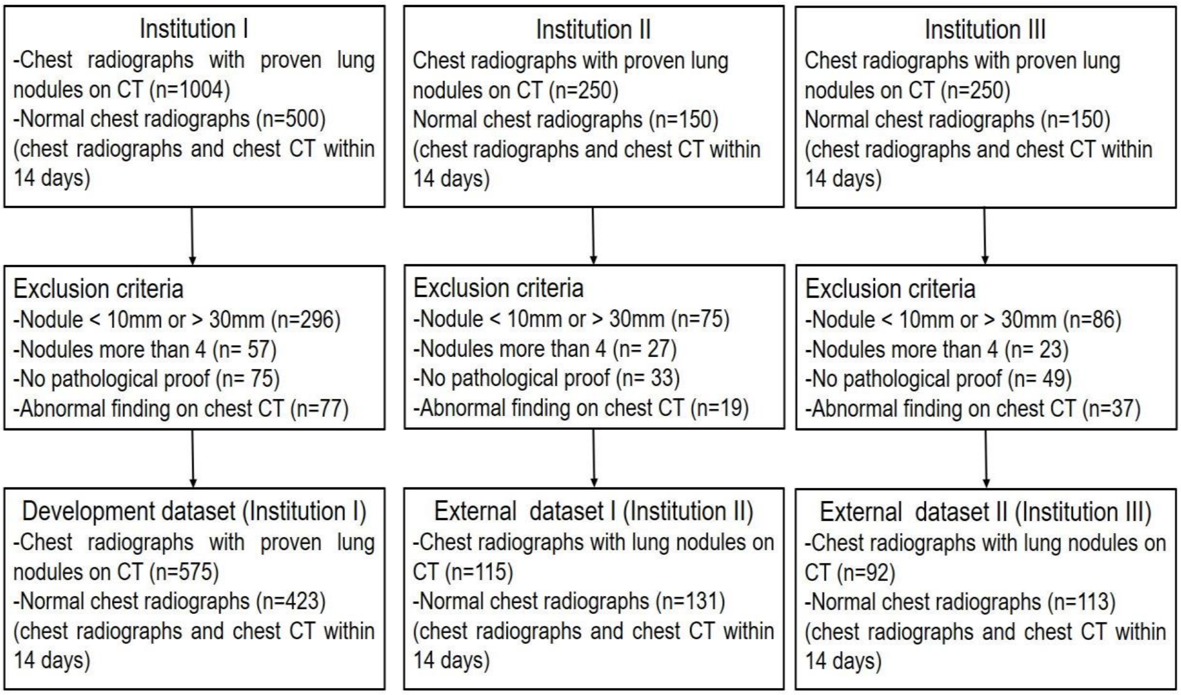

© 2023 Kim H et al. *JAMA Network Open.*

eFigure 2. The detailed architecture of deep learning–based synthetic bone–suppressed Image generator. The network was based on U-shaped encoder-decoder architecture and each part consisted of 8 hidden layers with skip connections.

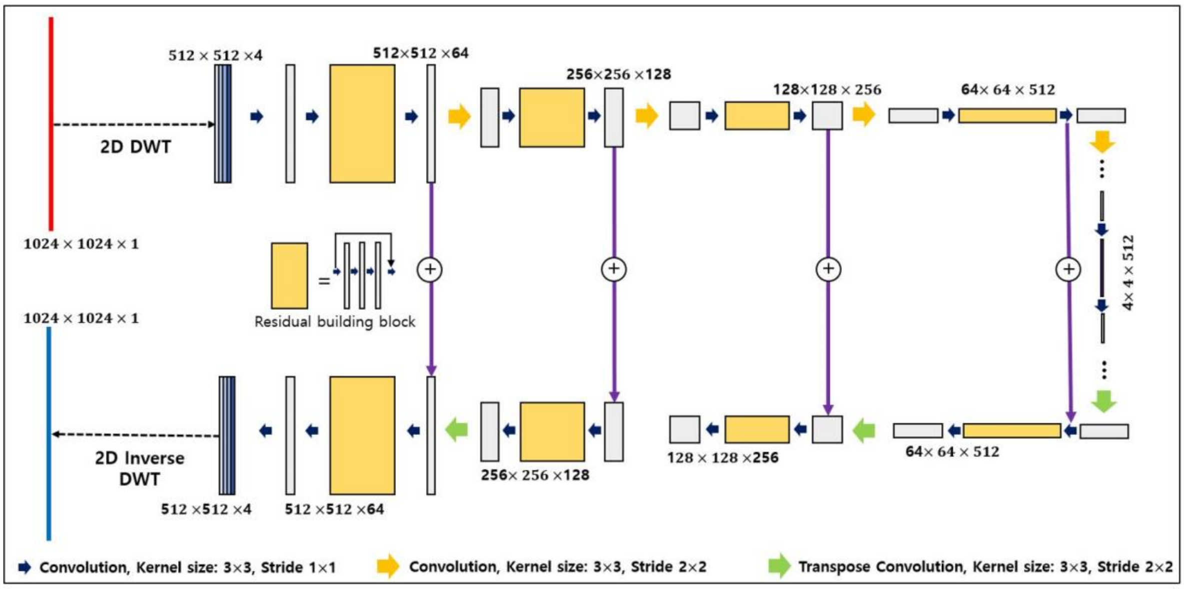

eFigure 3. Illustration of Representative Case of Nodule Detection Performance Between DLBS Model and CNN Model

eFigure 3A. Illustration of representative case of nodule detection performance between DLBS model and CNN model. On chest radiograph, the nodule was not detected by CNN model.

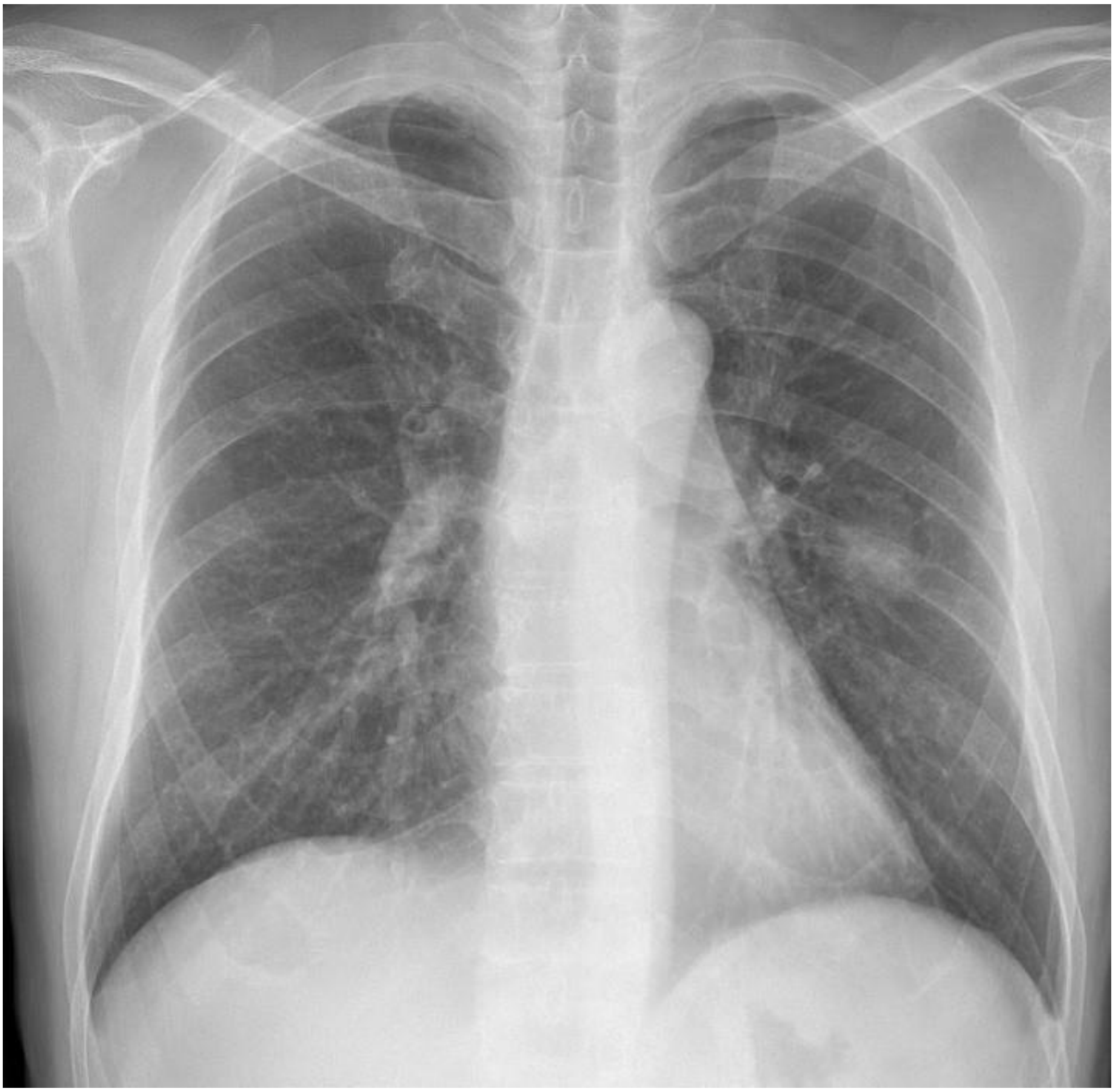

© 2023 Kim H et al. *JAMA Network Open.*

eFigure 3B. Illustration of representative case of nodule detection performance between DLBS model and CNN model. On bone-suppressed chest radiographs created using the DLBS model, the algorithm correctly detected the true nodule (red box: DLBS).

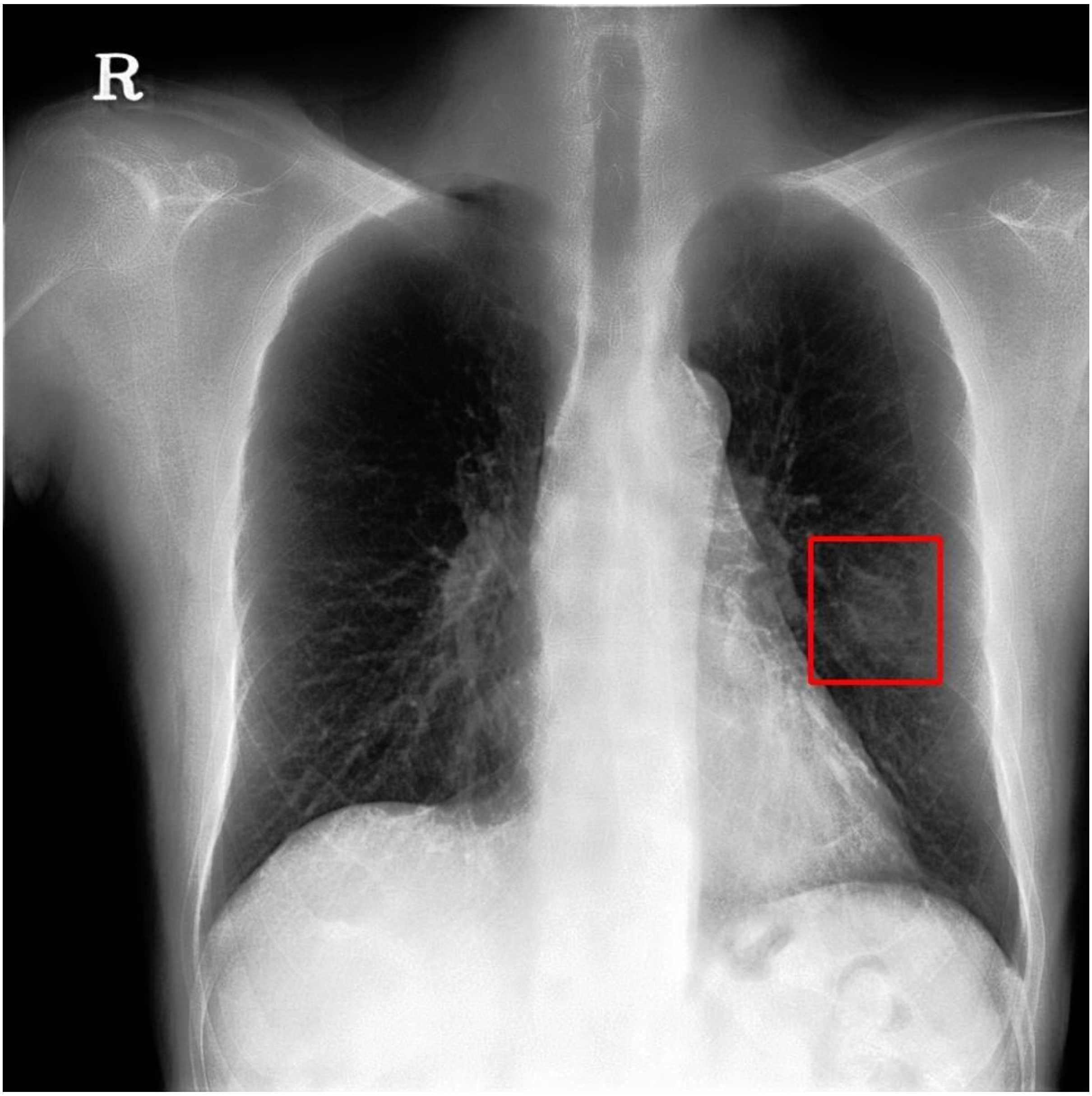

© 2023 Kim H et al. *JAMA Network Open.*

eFigure 3C. Illustration of representative case of nodule detection performance between DLBS model and CNN model. Chest CT examination revealed a 25-mm lung adenocarcinoma in the left lower lobe (arrow).

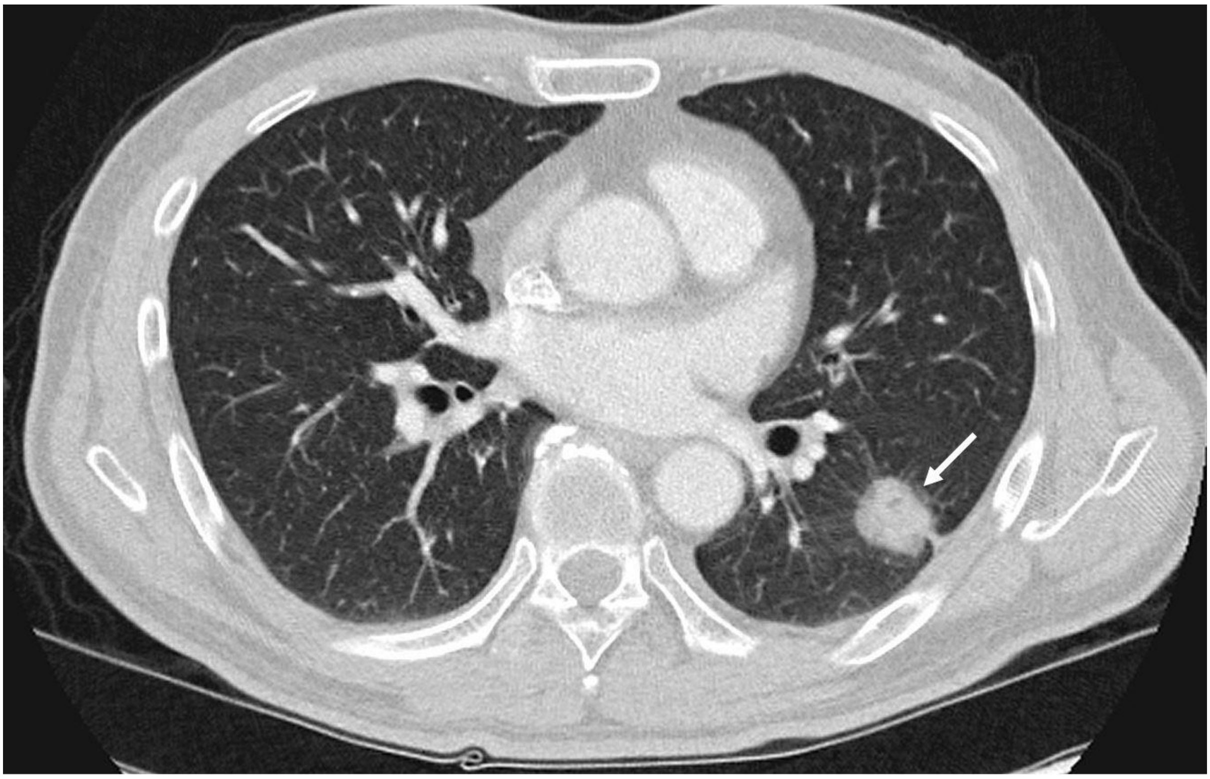

© 2023 Kim H et al. *JAMA Network Open.*
